# Quadratus Lumborum Block Versus Transversus Abdominis Plane Block for Postoperative Analgesia After Laparoscopic Colorectal Surgery

**DOI:** 10.3390/medicina61050825

**Published:** 2025-04-30

**Authors:** Mihaela Roxana Oliță, Mihai Adrian Eftimie, Bogdan Obrișcă, Bogdan Sorohan, Dragoș Eugen Georgescu, Liliana Elena Mirea, Dana Rodica Tomescu

**Affiliations:** 1 Anesthesiology and Intensive Care, Fundeni Clinical Institute, 022328 Bucharest, Romania; danatomescu@gmail.com; 2 Fundeni Clinical Institute, Carol Davila University of Medicine and Pharmacy, 050474 Bucharest, Romania; mihai.eftimie84@gmail.com (M.A.E.); obriscabogdan@yahoo.com (B.O.); bogdan.sorohan@yahoo.com (B.S.); llmirea@yahoo.com (L.E.M.); 3 Department of Surgery, Fundeni Clinical Institute, 022328 Bucharest, Romania; 4 Department of Nephrology, Fundeni Clinical Institute, 022328 Bucharest, Romania; 5 Department of General Surgery “I. Juvara”, “Dr. I. Cantacuzino” Clinical Hospital, 73206 Bucharest, Romania; 6 Anesthesiology and Intensive Care Clinic, Clinical Emergency Hospital Bucharest, 014461 Bucharest, Romania

**Keywords:** TAP block, QL block, opioid consumption, laparoscopic colorectal surgery

## Abstract

*Background and Objectives*: Extensive research has demonstrated that various approaches to the quadratus lumborum (QL) block offer superior postoperative analgesia compared to the transversus abdominis plane (TAP) block, particularly in reducing opioid consumption. This study aims to compare postoperative analgesia between the blocks in laparoscopic colorectal surgery. *Materials and Methods*: A retrospective analysis was performed on patients with elective colorectal surgeries who received bilateral US TAP blocks in the supine position or US anterior QL block in the lateral position at the end of the surgery and before extubating, with Ropivacaine 0.25%. Total opioid consumption and time to first intravenous analgesic were noted. *Results*: Between January 2020 and December 2024, 410 patients underwent elective laparoscopic colorectal oncology surgery under general anesthesia, with peripheral nerve blocks. Of these, we analyzed 116 patients with localized diseases who underwent elective surgeries and who did not require conversion to classical surgery and received either QL or TAP blocks. A total of 62 patients underwent QL block and 54 received TAP block. For the primary outcome, in the QL group, significantly fewer opioids were used than in the TAP group (*p* < 0.001), and time to first rescue analgesic was prolonged in the QL group at 16 h (IQR 14–18) compared to the TAP group, where the requirement occurred earlier at 8 h (IQR 8–8) postoperatively (*p* < 0.001). *Conclusions*: Postoperative bilateral US anterior QL block reduced morphine consumption and improved time to rescue analgesia and LOS compared with midaxillary line bilateral US TAP block.

## 1. Background

Colorectal cancer represents a significant global health challenge, ranking as the second most prevalent malignancy among women and the third among men, accounting for approximately 10% of all cancer diagnoses and related mortalities annually [1,2]. Surgical resection continues to be the cornerstone of curative management, frequently complemented by neoadjuvant or adjuvant chemotherapy and radiotherapy in carefully selected cases to enhance treatment outcomes.

Minimally invasive surgical techniques, particularly laparoscopic approaches, have become the gold standard for colorectal cancer treatment due to their well-established advantages, including reduced postoperative pain, shorter LOS, and faster recovery. These benefits are integral to enhanced recovery after surgery (ERAS) protocols, which aim to improve clinical outcomes and optimize recovery trajectories [3].

Effective postoperative pain management is a critical aspect of colorectal surgery, with multimodal analgesia serving as a cornerstone of contemporary pain control strategies. This approach incorporates regional anesthesia techniques, classified as either neuraxial (epidural analgesia) or peripheral blocks (TAP and QL blocks), combined with non-opioid analgesics to enhance pain relief while minimizing opioid requirements [4,5].

The TAP block has demonstrated significant analgesic efficacy in laparoscopic colorectal surgery, particularly during the first 24 to 48 h post operation [6]. This technique has been associated with reduced opioid consumption, improved pain scores, and the reduced use of nonsteroidal anti-inflammatory drugs (NSAIDs) [7]. The analgesic mechanism of the TAP block is attributed to the blockade of the anterior branches of the thoracolumbar nerves, specifically T6-T10 in the subcostal TAP block and T9-T12 in the posterior TAP block [8]. By delivering LA into the fascial plane between the IOM and TAM, the TAP block provides effective analgesia for the skin, musculature, and parietal peritoneum of the anterior abdominal wall, encompassing dermatomes T6 to L1 [9].

Visceral pain transmission occurs via the ventral branches of the spinal nerves, positioning the transmuscular quadratus lumborum (TQL) block as a potentially valuable technique for optimizing postoperative analgesia [10]. Initially described by Borglum et al., the TQL block involves the introduction of LA within the fascial plane between the PMM and the QLM [11].

The QL block has proven effective in abdominal surgery, regardless of whether the procedure is performed via open or laparoscopic approach [5,12]. This technique achieves a significant reduction in opioid consumption and an improvement in postoperative pain scores by delivering local anesthetic into the fascial plane between the PMM and QLM, as in the anterior QL block approach [13].

The primary aim of this study is to perform a comparison of the analgesic efficacy of the QL block versus the TAP block in patients undergoing elective laparoscopic colorectal surgery. The objective of this study is to assess and compare the efficacy of two techniques in managing postoperative pain and reducing opioid consumption within the first 48 h following surgery. This investigation also aims to identify whether the QL block, known for its potential to achieve more extensive and prolonged analgesia due to its deeper and more proximal distribution of LA, provides a clinically meaningful advantage over the TAP block, which primarily targets somatic pain within the anterior abdominal wall. Additionally, the secondary outcomes are represented by patient-reported pain scores at rest and during movement and time to first analgesic request. By elucidating these differences, this study analyzes perioperative pain management protocols, regional anesthesia techniques, and ultimately, recovery pathways for patients undergoing minimally invasive colorectal surgery.

## 2. Materials and Methods

### 2.1. Study Design and Ethical Approval

This monocentric study was conducted in a tertiary center, represented by the Oncological and Hepatobiliopancreatic Surgery Department of the Fundeni Clinical Institute, Bucharest, Romania. The study received ethical approval from the Fundeni Clinical Institute Ethics Committee (Study No. 1891/2025). Patient data were extracted exclusively from clinical records, ensuring the confidentiality and integrity of sensitive information. Data collection was performed by the anesthetic team, predominantly by anesthesiology residents under supervision.

### 2.2. Patients Selection

The study population included 410 adult patients, aged 18 to 90 years, who underwent elective laparoscopic oncological colorectal surgery, under general anesthesia, between January 2020 and December 2024. The procedures were performed by the same operating team from Fundeni Clinical Institute. In laparoscopic abdominal oncological surgery, peripheral nerve blocks are routine, and the type of block used is at the discretion of the attending anesthesiologist, who is part of a dedicated team, to ensure consistency in technique, procedural reliability, and subsequently, a uniform institutional environment.

#### 2.2.1. Inclusion Criteria

Only patients with complete and verifiable clinical records were included in the final analysis to support the integrity of the data review. The surgical indications and contexts were consistent across the population: all procedures were elective, and patients had been admitted prior to the day of surgery, underscoring the planned and controlled nature of perioperative management within this cohort. The application of peripheral nerve blocks was administered immediately at the end of surgery and before extubating, in accordance with the routine institutional practice at our tertiary oncological center.

#### 2.2.2. Exclusion Criteria

In order to ensure homogeneity among the patients analyzed, we used a series of exclusion criteria when collecting data from patient registries.

Clinical conditions that could influence nociception or analgesic efficacy were also considered. Patients with metastatic colorectal cancer or synchronous colorectal tumors were excluded, as were individuals with other concurrent malignancies capable of modifying pain perception. Those undergoing emergency surgical interventions for obstructive colorectal cancer or those presenting with severe anemia (hemoglobin < 7 g/dL) and associated hemodynamic instability were also excluded.

A history of major surgical procedures that could influence postoperative pain perception or regional anesthesia efficacy led to exclusion.

Finally, patients who required unplanned thoracic epidural analgesia or conversion from laparoscopic to open surgery intraoperatively were excluded post hoc, as such deviations from the study protocol could substantially impact the evaluation of analgesic techniques.

Based on the above-mentioned criteria, in our analysis, we included 116 patients who had undergone elective surgeries, who had localized diseases, and who did not require conversion to classical surgery and received either QL or TAP blocks.

### 2.3. Anesthesia and Surgical Procedures

General anesthesia was induced using intravenous propofol (1.5–2.0 mg/kg), fentanyl (5 µg/kg), and rocuronium (0.6 mg/kg). Maintenance involved sevoflurane, fentanyl boluses, and intermittent rocuronium dosing (0.1–0.2 mg/kg/h). Anesthetic depth was monitored with a bispectral index (BIS) maintained between 40 and 60. Intraoperative opioid administration (fentanyl and morphine) is performed generally at the discretion of the anesthesiologist but following department-wide dosage guidance.

Laparoscopic procedures were performed by the same surgical team using a standardized approach. Pneumoperitoneum was established and intra-abdominal pressure was maintained between 10 and 13 mmHg. Four to five laparoscopic ports were used, including a 1 cm incision for camera insertion.

### 2.4. Nerve Block Techniques

#### 2.4.1. Group QL Block (*n* = 62)

**Figure 1:**
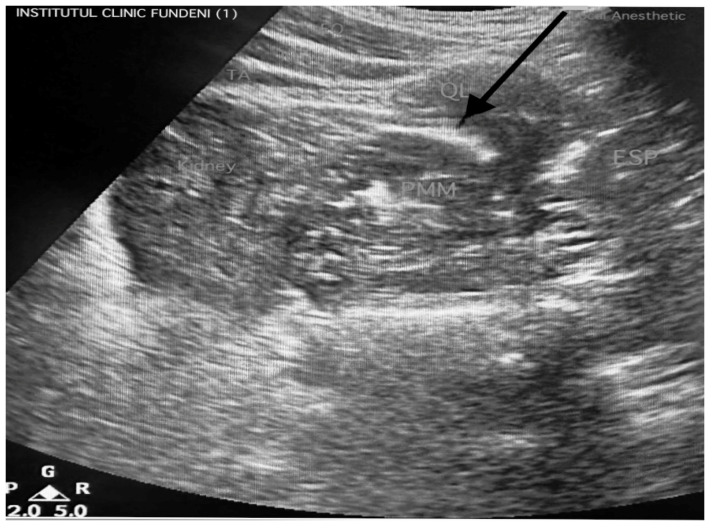
Ultrasound QL block approach: the needle is advanced in an anterolateral-to-posteromedial direction under US guidance to ensure precise localization. Bilateral type 3 (anterior) QL blocks were administered, with the injectate deposited at the fascial plane between the anterior border of the QLM and the PMM (from the authors’ collection). The arrow indicates the direction and the site of injection for the local anesthetic.

Anterior QL blocks were performed bilaterally under US guidance with 20 mL of 0.25% bupivacaine at each side after surgery. The block was administered in the lateral decubitus position at the L2–L3 vertebral level, identifying the “shamrock sign” (QLM, PMM, and ESM). The anesthetic was delivered in the fascial plane between the anterior QLM and the PMM. The procedure was replicated on the contralateral side to achieve symmetric analgesia. Successful block placement was confirmed through US documentation, indicating the spread of injectate within the target fascial plane, ensuring the adequate blockade of the relevant thoracolumbar nerves for optimal postoperative analgesia (Figure 1).

#### 2.4.2. Group TAP (*n* = 54)

**Figure 2:**
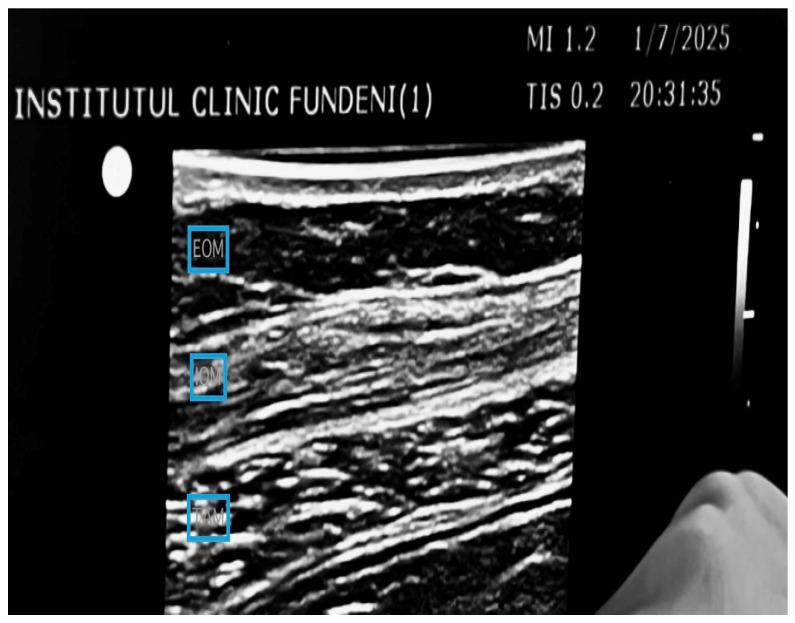
US TAP block approach: in the TAP group, the linear probe was placed in the transverse plane at the midaxillary line between the lower costal margin and iliac crest. When the EOM, IOM, and TAM were observed, a 20-gauge, 100 mm needle (Stimuplex^®^ Ultra 360^®^; B.Braun, Mihela Olita, Fundeni Clinical Institute, Bucharest, Romania, Affiniti 70, Philips Ultrasound from Fundeni Clinical Institute, Bucharest, Romania) was advanced using an in-plane technique in an anteromedial to posterolateral direction toward the TAP (the fascial plane between the IOM and TAM) (from the authors’ collection).

US TAP blocks were performed bilaterally under US guidance with 20 mL of 0.25% ropivacaine at each side after surgery. The needle was advanced within the fascial plane between the IOM and TAM. After checking the correct position of the needle tip, a dose of 20 mL of 0.25% ropivacaine was injected into the TAP. The TAP block was also applied bilaterally. A successful block was defined as the diffusion of anesthetic between the IOM and TAM (Figure 2).

### 2.5. Data Collection

The patient data, encompassing demographic characteristics, such as surgical information, analgesic regimens, and postoperative outcomes, were systematically obtained from observation charts. These are completed as routine during the perioperative period by a team largely made up of residents, whose direct and continuous involvement ensures adequate documentation and therefore minimizes the inter-observer variability.

The consistent maintenance of observation charts facilitated the accurate recording of critical parameters, enabling a reliable assessment of perioperative management strategies. This approach not only strengthened the validity of the collected data but also provided a robust foundation for evaluating the efficacy of analgesic techniques and their impact on postoperative recovery.

### 2.6. Pain Assessment and Analgesic Protocol

Postoperative pain scores and analgesic outcomes were analyzed based on clinical timepoints, as recorded in the anesthetic templates, from patient charts and electronic medical records. Pain intensity assessments are routinely documented, as per standard care policies in our center, at specific intervals—1, 2, 4, 8, 12, and 48 h—following the administration of the regional block, regardless of surgical approach. Pain assessments, as documented in charts, include evaluations at rest and during coughing. Initial pain scores are routinely recorded 1 h after patient extubation. Thereafter, subsequent evaluations were documented every two hours during the first 12 h and every four hours from the 12th to the 48th postoperative hour. These data were reviewed to evaluate the analgesic timeline of postoperative analgesia and to assess the effectiveness of the regional technique over time.

The duration of analgesia was defined as the time interval between the administration of the regional anesthetic and the first patient-initiated request for supplemental or rescue analgesia. As per institutional anesthetic protocol, regardless of the type and approach of surgical interventions, the rescue analgesia was administered in a departmental stepwise fashion: intravenous paracetamol was provided as the first-line agent upon documentation of a VAS score ≥ 3. If pain persisted and the VAS remained at ≥3 after two hours, intravenous diclofenac was administered at a dosage of 1.5 mg/kg, not exceeding 150 mg per 24 h period. In cases where pain control remained suboptimal, intravenous nefopam (maximum 60 mg per day) was subsequently used. For breakthrough pain unresponsive to these measures, intravenous morphine (0.1 mg/kg) was administered, based on clinical judgment.

The documentation of opioid consumption within the first 48 h, the total amount of non-opioid analgesics used, and the time to first rescue analgesia were available and recorded. These endpoints were selected to enable an accurate assessment of analgesic efficacy and the pharmacodynamic duration of the regional technique.

Adverse events are generally documented in the hospitalization index. For the analysis, particular emphasis was placed on the identification of bradycardia—defined as a heart rate of <60 beats per minute or a ≥20% reduction from baseline—and hypotension, defined as a ≥20% decline in baseline arterial pressure. The incidence and scheduling of these events were examined to assess the overall safety and tolerability of the analgesic method.

The primary endpoint was the total opioid consumption, measured from the time of anesthesia induction to the point of discharge from PACU.

Secondary endpoints included the time to first rescue analgesia, the quality of analgesia as determined by VAS at each timepoint (1, 2, 4, 8, 12, and 48 h, both at rest and during coughing), and the total consumption of intravenous non-opioid analgesics. Additional perioperative parameters included EBL, PACU stay, hospital stay, postoperative neutrophil to lymphocyte ratio as an early prognostic factor (defining the groups as <5% = low risk and >5% = high risk), and complications (according to the Clavien–Dindo classification).

### 2.7. Statistical Analyses

Continuous variables were summarized based on their distributional properties. For data adhering to a normal distribution, descriptive statistics were expressed as the mean accompanied by the standard deviation or the 95% confidence interval. In cases where data deviated from normality, the median was reported alongside the interquartile range (IQR), which represents the 25th to 75th percentiles. The assessment of normality was conducted using the Shapiro–Wilk test. Categorical variables were presented as absolute frequencies and percentages, reflecting the distribution of each category within the study cohort. To evaluate differences between groups, appropriate statistical tests were employed based on the nature and distribution of the variables. For continuous variables, Student’s *t*-test was utilized to compare means between two groups when the data were normally distributed. Conversely, the Mann–Whitney test was applied for non-normally distributed data. When comparing means across multiple groups, one-way analysis of variance (ANOVA) was used for normally distributed data, while the Kruskal–Wallis test served as the non-parametric alternative for data that did not meet normality assumptions.

For repeated measurements of pain intensity, as assessed using the VAS at multiple postoperative timepoints, data were analyzed using a repeated measures ANOVA for normally distributed data or the Friedman test for non-parametric data.

Differences in categorical variables between groups were evaluated using the Pearson chi-square test. In instances where sample sizes were small or the assumptions of the chi-square test were not met, Fisher’s exact test was employed.

All statistical analyses were performed using IBM SPSS Statistics, version 29.0.0 (IBM Corp., Armonk, NY, USA), and the graphs were developed using GraphPad (GraphPad Prism, 10.4.1). In all analyses, *p*-values were two-tailed, and a *p* < 0.05 was considered statistically significant.

## 3. Results

### 3.1. Study Population

The characteristics of the study population are described in Table 1. The analysis included 116 adult patients (65.5% males) who underwent elective laparoscopic oncological colorectal surgery and peripheral nerve blocks at the end of the surgery. Among them, 62 patients received a quadratus lumborum (QL) block and 54 patients had undergone a transversus abdominis plane (TAP) block.

Comparing the two groups of anesthetic blocks, similarities were identified in terms of demographics and clinical status at baseline. The mean age across the full cohort was 67.8 ± 8.6 years, with no statistically significant difference between the TAP group (68.1 ± 9.4 years) and the QL group (67.7 ± 7.9 years; *p* = 0.8). This reflects a population of predominantly older adults, which aligns with the typical demographic profile of patients undergoing colorectal cancer surgery.

In terms of nutritional and metabolic profile, patients in both groups were generally overweight, with a median body mass index (BMI) of 29.0 kg/m^2^ (IQR: 27.2–31.7). The TAP and QL groups exhibited similar BMI distributions (TAP: 29 [27.7–31.0]; QL: 29.5 [27.0–32.0]), with no indication of significant metabolic disparity at baseline.

Perioperative risk status, as assessed by the American Society of Anesthesiologists (ASA) classification, was also balanced across groups. Most patients were categorized as ASA II or III, indicating the presence of mild to moderate systemic disease. Specifically, 46.6% of the total cohort were ASA II (TAP: 46.3%, QL: 46.8%), and 53.4% were ASA III (TAP: 53.7%, QL: 53.2%), with no statistically significant intergroup variation (*p* > 0.59).

### 3.2. Surgical Characteristics

Within the analysis, operative variables were examined to evaluate potential differences in surgical complexity and intraoperative outcomes between the two patient cohorts: those receiving TAP blocks and those managed with QL blocks. All surgical procedures were performed using a minimally invasive laparoscopic approach, by an experienced surgical team, thereby ensuring procedural consistency across the study population.

With respect to surgical indications and procedure type, the distribution across groups demonstrated some variability. The most performed procedure overall was low anterior resection, comprising 53.7% of interventions in the TAP group and 27.4% in the QL group, contributing to 39.7% of all procedures. This disproportion may partially account for the increased EBL and prolonged operative time observed in the TAP cohort. Left-sided colonic resections were comparably distributed (TAP: 29.6%, QL: 32.2%), while subtotal colectomies were more frequently performed in the QL group (22.6%) than in the TAP group (7.4%). Right-sided colonic resections (TAP: 3.7%, QL: 9.7%) and stoma creation (TAP: 5.6%, QL: 8.1%) were less frequent overall and relatively evenly allocated between the two arms. Taken together, the surgical profiles of the TAP and QL groups reflected real-world heterogeneity in oncological colorectal resections. However, the laparoscopic approach performed by the same surgical team allowed for consistent technique, thereby strengthening the reliability of outcome comparisons. The significantly lower EBL in the QL group warrants further exploration as a potential clinical advantage of this technique in similar surgical context (Table 2).

The median operative time was 240 min (IQR: 190–300) for the TAP group and 210 min (IQR: 190–267.5) for the QL group. Although this observed difference may reflect procedural complexity or patient-specific anatomical considerations, statistical analysis revealed no significant difference in surgical duration between the two groups (*p* = 0.3).

A more pronounced intergroup difference was noted in terms of EBL. Patients in the TAP group experienced a median EBL of 175 mL (IQR: 115–300), whereas those in the QL group exhibited significantly lower volumes, with a median of 100 mL (IQR: 92.5–100). This difference reached statistical significance (*p* < 0.001) and may reflect either inherent group differences in surgical burden or differential efficacy of intraoperative hemostasis, although all procedures were carried out under standardized surgical conditions.

The analysis of postoperative analgesic consumption revealed significant differences between the QL and TAP groups. Opioid consumption (mg) within the first 24 h post operation was notably lower in the QL group compared to the TAP group (12 mg vs. 18 mg, respectively; *p* < 0.001). Equivalent opioid consumption was calculated based on the administration of fentanyl or morphine during and after the surgical procedure. Similarly, equivalent non-opioid consumption, expressed as a percentage (%) of the daily maximum allowance (as regulated by the National Drug Agency), was also significantly reduced in the QL group. This included the cumulative use of acetaminophen, diclofenac sodium, and nefopam hydrochloride. All data were reported as median values with interquartile ranges (IQR) (Table 3).

**Figure 3:**
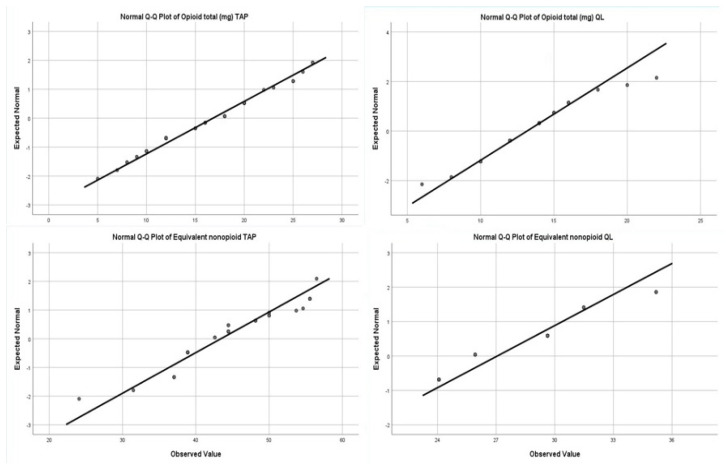
Q-Q plot reflects total opioid consumption in the first 24 h between the two groups with a median 12 mg in the QL group vs. 18 mg in the TAP block. *p* < 0.001 and Q-Q plot for equivalent non-opioid consumption was lower in the QL group vs. the TAP group. *p* < 0.00. In the QL group, the requirement for first non-opioid intravenous was 16 h with a median of 14–18, reduced significantly as compared to the TAP group’s block, which also offers adequate analgesia, with the need for the earlier administration of non-opioid intravenous analgesia, respectively, at 8 h post operation, *p* < 0.001.

Primary outcome data were assessed for normality with Shapiro–Wilk tests and inspected with Q-Q plots: opioid consumption for TAP (*p* = 0.064) and QL (*p* = 0.002) and non-opioid consumption for TAP (*p* = 0.000) and QL (*p* = 0.000). Consequently, Mann–Whitney rank sums method was chosen to compare the opioid consumption in the TAP and QL groups, concluding that the QL block is more efficient for pain control as reflected by a lower opioid consumption (two-tailed *p* = 0.000). Non-opioid consumption was compared in the two groups using the same method with similar results (two-tailed *p* = 0.000), which is a further argument for QL superiority in pain control as compared to TAP (Figure 3).

**Figure 4:**
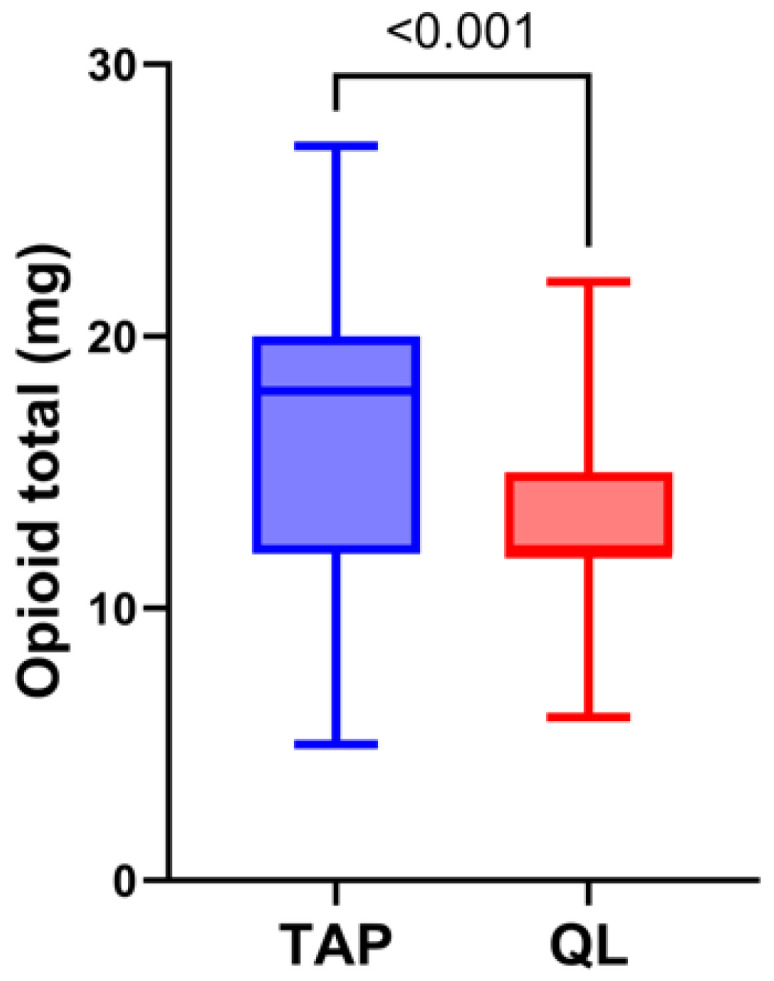
Opioid consumption. The figure illustrates a significant reduction in cumulative opioid consumption within the first 24 h post operation in patients receiving a quadratus lumborum (QL) block compared to those with a transversus abdominis plane (TAP) block.

In our analysis, in the first 24 h post surgery, total opioid consumption was significantly lower in the QL group (13 ± 2.6 mg) compared to the TAP group (16.8 ± 5.5 mg), and it was observed that patients in the QL group had a median time of 16 h (IQR 14–18) to the first administration of non-opioid intravenous analgesia. In contrast, patients in the TAP group received their first dose significantly earlier, with a median time of 8 h (IQR 8–8) post operation (*p* < 0.001, Figure 4).

**Figure 5:**
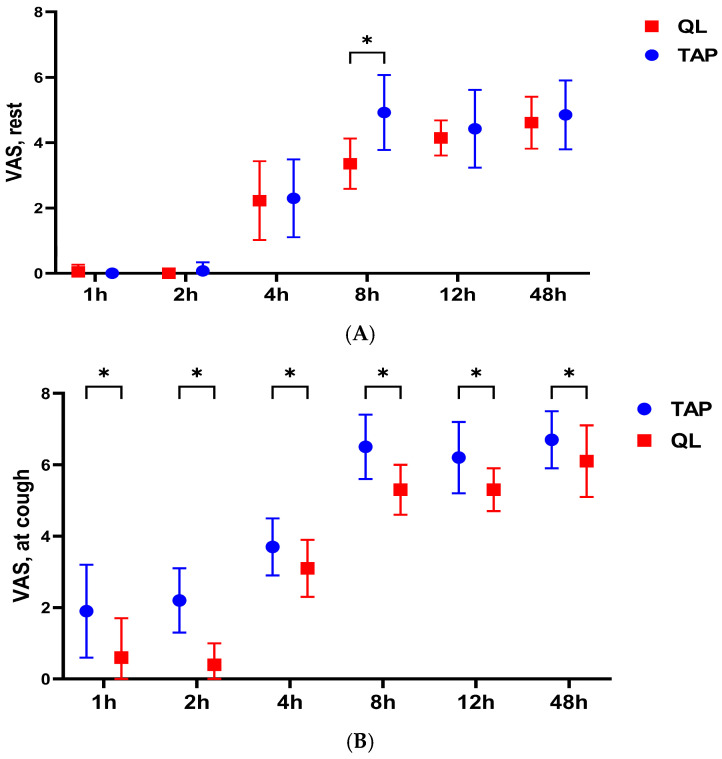
Pain scores (VAS) at rest and during coughing effort in the first 48 h post operation. (**A**): VAS at rest. (**B**): VAS during coughing. Data are presented as median, range, and interquartile range. * *p* < 0.001. QL: quadratus lumborum; TAP: transversus abdominis plane; VAS: visual analog scale.

Postoperative pain score analysis demonstrated that VAS at rest was reduced in both groups; however, at 8 h post block, the scores remained significantly lower in the QL group (*p* < 0.001). Furthermore, a statistically significant difference was observed in VAS during coughing efforts at 1, 2, 4, 8, 12, and 48 h post operation, with the QL block showing superior analgesic efficacy (*p* < 0.001, Figure 5A,B).

The analysis revealed significant reductions in LOS (days) in the QL group compared to the TAP group. Specifically, the LOS decreased from 3 days in the TAP group to 2 days in the QL group (*p* < 0.001), and the total hospitalization duration was reduced from 7 days in the TAP group to 5 days in the QL group (*p* < 0.001). These findings highlight statistically meaningful improvements in postoperative recovery metrics associated with the QL intervention.

To further explore the relationship between the type of block and postoperative outcomes, a chi-square test of independence was performed to assess the association between the type of block (QL vs. TAP) and the postoperative neutrophil-to-lymphocyte ratio (NLR), an early prognostic factor. Patients were stratified into two groups based on NLR: <5% (low risk) and >5% (high risk). The analysis demonstrated a significant relationship, χ^2^ (1, *n* = 116) = 44.888, *p* = 0.000, indicating that patients in the TAP group were more likely to fall into the high-risk NLR category, which was associated with increased LOS and prolonged time to discharge, compared to the QL group.

Both the QL and TAP techniques demonstrated a favorable safety profile, with no occurrence of major postoperative complications. According to the Clavien–Dindo classification, no complications ≥3 were observed in either the QL or TAP groups.

## 4. Discussion

The results showed that the QL block provides more effective analgesic relief compared to the TAP block in the postoperative period, after laparoscopic colorectal surgery. This suggests that utilizing the QL block may enhance postoperative pain management and patient comfort.

The analysis showed that the US-guided anterior QL block manages pain during postoperative stages of surgical procedures, through decreased opioid consumption in first- and second-day scores and the pain scores at 8 h, 12 h, and 48 h.

The results of this study support the effectiveness of multimodal analgesia in reducing opioid consumption during the postoperative recovery phase of laparoscopic colorectal surgery. Moreover, our findings suggest that the US-guided QL block is more efficacious than the US-guided TAP block in providing postoperative pain relief following laparoscopic colorectal surgery. These findings are consistent with those of Deng et al., who demonstrated that the QL block is associated with superior postoperative analgesia, as evidenced by a significant reduction in fentanyl consumption at both 24 and 48 h post operation [14].

A RCT conducted by Huang, D., et al. in 2020 validated the effectiveness of the QL block and demonstrated a reduction in opioid consumption compared to that in patients with a TAP block. This study indicates that patients who received a QL block consumed significantly less morphine [15]. The incorporation of the QL block into multimodal analgesic regimens for laparoscopic colorectal surgery is supported by robust evidence, highlighting its efficacy in achieving superior postoperative pain control, reducing opioid in pain relief, and improving accelerated postoperative recovery, as was highlighted in a review by Malla et al., involving 188 patients, which concluded that the QL block is generally a more preferable regional technique than the TAP block [16,17].

Preliminary studies investigating the application of the QL block in upper abdominal procedures, such as laparoscopic cholecystectomy, gastrectomy, and hepatic resections, suggest its efficacy in reducing postoperative pain [18,19,20,21,22]. This is likely due to the cephalad spread of LA into the thoracic paravertebral space, which allows the coverage of dermatomes T7–T12, thereby addressing both visceral and somatic pain pathways [11,23]. The use of the QL block in surgeries involving both upper and lower abdominal regions remains an area of limited exploration. Anatomical and clinical data indicate that the efficacy of the block depends on factors such as the approach utilized (e.g., anterior QL1, posterior QL2, or transmuscular QL3) [24,25], the volume of LA administered, and patient-specific anatomical variability [26].

Postoperative pain management in laparoscopic colorectal surgery presents unique challenges due to the complex interplay of visceral pain and somatic pain during the acute recovery phase [27,28]. An understanding of these pain mechanisms, their clinical manifestations, and appropriate management strategies is critical for optimizing patient outcomes, particularly within the framework of enhanced recovery after surgery (ERAS) protocols [29].

Postoperative pain assessment using the numeric rating scale (NRS) and visual analog scale (VAS) shows strong correlations. These methods complement each other in measuring pain intensity, but individual patient preferences and clinical context should guide tool selection [30,31,32].

Preoperatively, NLR serves as an indicator of the balance between tumor progression and immune function, with elevated values often associated with increased tumor proliferation, metastatic potential, and reduced immune activity. Postoperatively, NLR reflects the stress response, providing insight into the body’s inflammatory state and its capacity for recovery [33].

These findings highlight the utility of NLR and PLR as practical and informative markers for assessing perioperative inflammation and stress. The elevated postoperative NLR observed in this study underscores its potential as a prognostic tool, particularly in identifying patients at risk of adverse outcomes. Further investigation is warranted to elucidate the interplay between anesthesia, surgical trauma, and these hematologic parameters, as well as their broader implications for perioperative management and patient care [34]. Despite the identification of numerous biomarkers for inflammation, none have consistently demonstrated capacity for the early, specific, and accurate diagnosis of systemic inflammation, but declining levels of butyrylcholinesterase (BChE) on the first and third postoperative days are significantly associated with an elevated risk of surgical site infections. BChE has recently gained recognition as a promising diagnostic marker for low-grade systemic inflammation [35].

The potential limitations are mainly represented by the retrospective and monocentric analysis of the study. Also, the absence of randomization of the patients to the QL or TAP block groups introduces a potential risk of selection bias. The choice of analgesic technique may have been influenced by clinical judgment or provider preference, which could have contributed to inherent differences between the groups and confounded the observed outcomes. Furthermore, the heterogeneity in surgical profiles, despite the use of the same surgical approach (laparoscopic), adds another layer of complexity when interpreting the results. Finally, the specific exclusion criteria, while intended to enhance homogeneity, may limit the generalizability of the findings to a broader patient population.

These limitations highlight the importance of cautious interpretation and underscore the need for future prospective, randomized controlled trials to validate these results. Looking ahead, such studies will be crucial in advancing our understanding and confirming these findings.

## 5. Conclusions

The QL block demonstrated superior analgesic efficacy compared to the TAP block, as indicated by a prolonged pain-free interval prior to block resolution, reflected in the delayed administration of the first intravenous analgesic. Additionally, the QL block was linked to a substantial decrease in both opioid and non-opioid analgesic consumption—each of which was reduced by roughly one-third—and a shorter length of hospital stay (LOS), resulting in an accelerated discharge timeline.

## Figures and Tables

**Table 1:** Population description.

Variable	TAP Block (*n* = 54)	QL Block (*n* = 62)	Total (*n* = 116)	*p*-Value
Female—*n* (%)	21 (38.9%)	19 (30.6%)	40 (34.5%)	–
Male—*n* (%)	33 (61.1%)	43 (69.4%)	76 (65.5%)	–
Age (years, mean ± SD)	68.1 ± 9.4	67.7 ± 7.9	67.8 ± 8.6	0.8
BMI (median, IQR)	29 (27.7–31.0)	29.5 (27.0–32.0)	29 (27.2–31.7)	–
ASA II—*n* (%)	25 (46.3%)	29 (46.8%)	54 (46.6%)	0.96
ASA III—*n* (%)	29 (53.7%)	33 (53.2%)	62 (53.4%)	0.59

Abbreviations: SD, standard deviation; ASA: American Society of Anesthesiologist; BMI: body mass index; QL: quadratus lumborum; TAP: transversus abdominis.

**Table 2:** Surgical characteristics.

Variable	TAP Block (*n* = 54)	QL Block (*n* = 62)	Total (*n* = 116)	*p*-Value
Surgical time—min	240 (190–300)	210 (190–267.5)	230 (190–287.5)	0.3
Bleeding—mL	175 (115–300)	100 (92.5–100)	100 (100–200)	<0.001
Low anterior resection	29 (53.7%)	17 (27.4%)	46 (39.7%)	–
Right side colonic	2 (3.7%)	6 (9.7%)	8 (6.9%)	–
Left side colonic resection	16 (29.6%)	20 (32.2%)	36 (31.0%)	–
Subtotal colectomy	4 (7.4%)	14 (22.6%)	18 (15.5%)	–
Creation of a stoma	3 (5.6%)	5 (8.1%)	8 (6.9%)	–

Abbreviations: min: minutes; mL: milliliters; QL: quadratus lumborum; TAP: transversus abdominis plane.

**Table 3:** Primary outcomes.

Outcome	TAP Block (*n* = 54)	QL Block (*n* = 62)	*p*-Value
Time to first iv analgesic (h)	8 (0)	16 (14–18)	<0.001
Equivalent opioid consumption (mg)	18 (8)	12 (2.8)	<0.001
Equivalent non-opioid consumption (%)	40.8 (9.3)	25.9 (5.5)	<0.001

Abbreviations: h: hours; mL: milliliters; mg: milligrams; QL: quadratus lumborum; TAP: transversus abdominis plane.

## Data Availability

The raw data supporting the conclusions of this article will be made available by the authors on request.

## Abbreviations

EOM—External Oblique Muscle; IOM—Internal Oblique Muscle; LA—Local Anesthetic; PACU—Post Anesthesia Care Unit; QLM—Quadratus Lumborum Muscle; TAM—Transversus Abdominis Muscle; TAP—Transversus Abdominis Plane; VAS—Visual Analog Scale; ESM—Erector Spinae Muscle; PMM—Psoas Major Muscle; US—Ultrasound; EBL—Estimated Blood Loss; LOS—Length of Stay.
